# Hypoglycaemia without diabetes encountered by emergency medical services: a retrospective cohort study

**DOI:** 10.1186/s13049-018-0480-7

**Published:** 2018-02-01

**Authors:** Hanna Vihonen, Markku Kuisma, Jouni Nurmi

**Affiliations:** 1 Department of Emergency Medicine and Services, Päijät-Häme Central Hospital, Keskussairaalankatu 7, 15850 Lahti, Finland; 2 Department of Emergency Medicine, Helsinki University and Helsinki University Hospital, Lahti, Finland

**Keywords:** Glucose, Hypoglycaemia, Emergency medical service, Without diabetes

## Abstract

**Background:**

The current study investigates the incidence, aetiology, and outcome of hypoglycaemia of patients without diabetes in the EMS.

**Methods:**

The study was a retrospective cohort study that utilized electronic EMS patient record system (population of one million). All patients encountered by EMS with plasma glucose ≤3.9 mmol/l from 2009 to 2015 were included in the study and hospital records were screened manually to detect possible reasons for hypoglycaemia. Data from the governmental health insurance agency for all residents in Finland was used to reveal the diabetes status of the patients. Survival of the patients was followed from Population register centre up to six years. Serious hypoglycaemia was defined as plasma glucose ≤3.0 mmol/l.

**Results:**

From EMS cases with a plasma glucose measurement a total of 5467 hypoglycaemic patients without diabetes were encountered by EMS during the study period with an incidence of 1082 (CI95% 1019–1148) per 100,000 inhabitants per year, corresponding 41.6%, (CI95% 40.8–42.3) of all hypoglycaemic patients. Of those patients, 3856 [71.6%, (CI95% 70.4–72.8)] were transported to hospital and 910 [23.2%, (CI95% 22.0–24.6)] had serious hypoglycaemia. The three main diagnosis groups that appeared in the subsequent hospital treatment associated with hypoglycaemia in all transported cases without diabetes as well with serious hypoglycaemia cases were: alcohol abuse [41.2%, (CI95% 39.7–42.8) and 42.2%, (CI95% 39.0–45.4)], hypothermia [17.2%, (CI95% 16.0–18.4) and 27.4%, (CI95% 24.6–30.4)], and malnutrition [16.9%, (CI95% 15.8–18.1) and 25.1%, (CI95% 22.4–28.0)]. Mortality ranged from 0.6–65.4% depending of admission reason and increased significantly at long-term. Non-Diabetics survival was less than with diabetics, when serious hypoglycaemia was present.

**Discussion:**

The most common possible hypoglycaemia related aetiological causes encountered in the EMS, alcohol abuse, hypothermia, and malnutrition, although frequent are often relatively benign conditions. These possible causes of hypoglycaemia can often be treated at scene or need only short hospital admissions. Hence they are not so prevalent in hospital studies.

**Conclusions:**

Hypoglycaemia without diabetes is commonly observed among the hypoglycaemic EMS cases. Main causes for it are alcohol abuse, hypothermia, and malnutrition. Mortality correlated with age, higher priority dispatch codes, and plasma glucose rate in multivariate logistic regression analysis. Some of the etiological subgroups carry a markedly high mortality rate.

**Electronic supplementary material:**

The online version of this article (10.1186/s13049-018-0480-7) contains supplementary material, which is available to authorized users.

## Background

Plasma glucose disturbances are commonly observed in the emergency medical services (EMS) setting. Most of the hypoglycaemic events are related to treatment of diabetes. However, hypoglycaemia is frequently also observed in patients without diabetes [1]. Hypoglycaemia in patients without diabetes has been supposed to be associated with a high mortality rate [2, 3]. In hospitalized patients without diabetes, the risk of developing hypoglycaemia is associated with malnutrition, malignancy, renal disease, congestive heart failure, and sepsis [4].

The causes of hypoglycaemia in patients without diabetes in the EMS patient population or the outcome of these patients are not well known. Based on our clinical observations, we hypothesized that EMS encounters even more cases of hypoglycaemia than previously reported in hospital patient studies [3], and that hypoglycaemia is commonly a marker of severe illness with poor prognosis. The aim of our study was to describe the incidence, aetiology and outcome of hypoglycaemia in patients without diabetes in the EMS.

## Methods

### Study design

We conducted a retrospective cohort study based on the electronic EMS records and hospital patient records combined with national registry data on cause of death and reimbursement entitlement status of medicine. Survival of patients was followed up to six years. The study protocol was approved by Helsinki University Hospital and no ethical board approval was needed because of retrospective design and as only registry data was used.

### Population

We included all patients in the Helsinki University Hospital area encountered by EMS and with measured hypoglycaemia during the years 2009 to 2015. The hypoglycaemia was defined as plasma glucose ≤3.9 mmol/l, in accordance with definitions by European medicines agency (EMA) and American diabetes association (ADA) [5, 6]. We also studied separately the serious hypoglycaemia group of plasma glucose ≤3.0 mmol/l accordance with ADA and European Association for the Study of Diabetes [7].

### Study setting and data

Helsinki University Hospital has the responsibility to organize and supervise EMS for about one million inhabitants in the Helsinki metropolitan area. The EMS consists of three fire and rescue departments and three private ambulance companies. They all use the same electronic patient record system (Merlot Medi®, CGI, Finland). Patients are typically transported to the six receiving public hospitals in Helsinki area, when necessary. If the patient does not require further medical treatment immediately the patient may be left at the scene and referred to other services, as necessary.

Paramedics measured plasma glucose values mainly from capillary samples using a plasma calibrated analysing device (e.g., the Optimum Xceed glucometer and MediSense Optimum electrodes manufactured by Abbott Laboratories, Alameda, CA). However, a small number of samples may have been venous whole blood draws, as this EMS system does not maintain a standard operating procedure for drawing blood. Plasma glucose was measured according to local protocol, which included patient cases with the following: lowered level of consciousness, sudden deterioration of overall wellness from an unknown cause, disorientation or agitation, seizure, a diabetic patient, who is feeling unwell or hypothermia. EMS personnel interviewed the patient or next of kin for current medical condition and gathered information form the social security card, which holds codes for conditions such as hypertension, renal failure, ischemic heart condition, congestive heart failure, and neurologic disorders. The causes of hospital stay and diagnoses that potentially are associated with hypoglycaemia were collected manually from hospital records. An electronic patient record system is used in the hospitals. Diabetes was determined by Social Insurance Institution Finland’s antihyperglycaemic medication reimbursement entitlement records. A record of prior purchase of antihyperglycaemic medication during the hypoglycaemic event confirmed the patient to have diabetes. Additional 89 cases of dietary, pregnancy related, and foreigners without antihyperglycaemic reimbursement entitlement medication records in Social Insurance Institution of Finland were added to the data manually from hospital patient record data. All other patients where considered to be patients without diabetes. Survival of the patients was studied up to six years using Statistics of Finland registry office data.

As blood glucose was measured mainly form capillary samples, with authors discretion as one of possible hypoglycaemia related condition, we considered such cases as acute pain, panic-attack, acute bleeding, anaphylaxis, and dyspnoea, where acute sympathetic nervous system activation and subsequent peripheral vasoconstriction of blood vessels were present to be pseudohypoglycaemic cases as no systemic hypoglycaemia was likely present [8].

### Data analysis

Electronic EMS records were studied for chronic medical conditions for comparison of diabetics and non-diabetics. For patients without diabetes, we searched the patient records for the hospital treatment and admission cause that might be related to the development of hypoglycaemia. Mortality data was collected from Statistics of Finland, the National registry office. At time of data collection statistics of years 2009–2014 were available. For Kapplan-Mayer survival analyses, the patients were divided into groups based on the seriousness of hypoglycaemia and diabetic status. Patients that were treated on scene by EMS were excluded from the study due to lack of accurate etiological data records.

### Possible aetiological causes

The possible aetiological causes in this study were defined by previously known possible hypoglycaemia related causes from the literature and by author’s discretion (pseudohypoglycaemic cases). If hospital records during hospital stay mentioned patients suffering from renal failure, liver failure, congestive heart failure, endocrinological causes, malignancies, cardiac arrest, current infections, current alcohol abuse, current intoxication, indication of acute or chronic malnutrition, patient suffering from a neurologic disorder (epilepsy, stroke, brain injury, brain haemorrhage, Parkinson’s disease), or the body temperature was mentioned to be under 36°C degrees, which was considered as hypothermia, these were marked as a possible association of hypoglycaemia. Often more than one possible aetiological cause may have caused a hypoglycaemic episode. If the possible aetiological cause was mentioned in the hospital records during the hospital stay after the hypoglycaemic episode was encountered by EMS and transported to hospital, we included it in the results. Hence possible hypoglycaemia related causes overlapped as concomitant causes on each other with no main cause. In some case the hospital records had only one possible cause that was possibly related to a hypoglycaemia episode. To clarify if there was any bias in the results we studied also the aetiology of these cases separately in Additional file 1.

Continuous variables were analysed using an unpaired t-test and the Man-Whitney test. Skewed variables are presented as median and inter-quartile range (IQR). Categorical variables were presented as percentages and analysed by Chi-square test. Univariate and multivariate logistic regression analysis were used to calculate odds ratios for mortality. Kapplan-Mayer survival curves were plotted for survival analysis and Log-rank test was used to calculate correlation. *P*-value < 0.05 was considered statistically significant. All statistical analyses were carried out using Graph Pad 7.0 for MAC OS X (Graph Pad Software, San Diego, CA, USA) and SPSS 23 for MAC OS X.

## Results

**Fig. 1:**
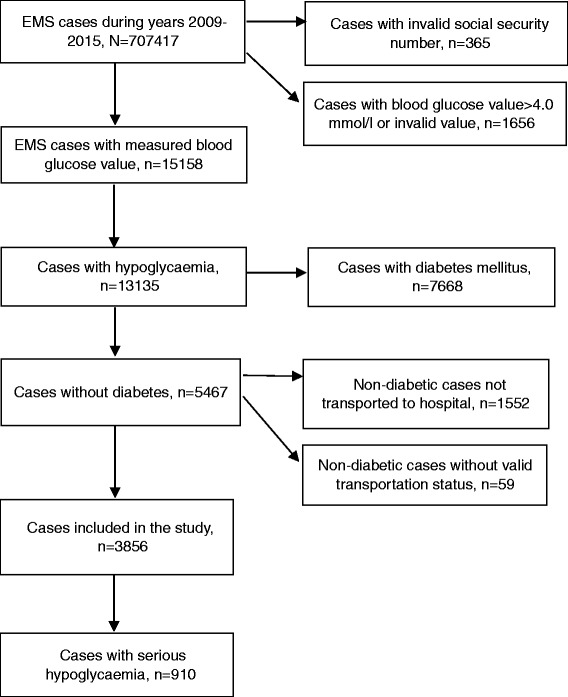
Inclusion and exclusion of study patients

A total of 13,135 hypoglycaemia episodes from 505,180 EMS cases were recorded during the seven-year study period, which was 4,5% of all EMS cases with a plasma glucose measurement. The final data included 3856 transported hypoglycaemic events without diabetes, which were analysed further (Fig. 1). Thus, the incidence rate of EMS-encountered hypoglycaemic patient cases without diabetes was 1082 (CI95% 1019–1148) per 100,000 inhabitants per year. Comparison of demographics, chronic medical conditions, transportation, vital parameters, and overall mortality rate between patients with diabetes and without diabetes as well as all patients is shown in Table 1.

**Table 1:** Demographics, vital signs, previous comorbidities, transportation, and overall mortality rate of patient cases with diabetes and without diabetes

	All patients (*N* = 13,135)	Patients with diabetes (*N* = 7668)	Patients without diabetes (*N* = 5467)
Age (years)	54, (38–67)	57, (44–68)	48, (30–63)
Age ≥ 65 year (%)	28, (28–29)	33, (32–34)	22, (21–24)
Sex, male (%)	63.4, (62.6–64.2)	65.2, (64.2–66.3)	60.8, (59.5–62.1)
Systolic blood pressure (mmHg)	136, (120–153)	139, (123–158)	132, (116–148)
Temperature (°C)	36.3, (35.5–36.8)	36.0, (35.2–36.5)	36.5, (36.0–37.1)
Blood oxygen saturation, median (%)	97, (95–99)	97, (95–99)	97, (95–99)
Heart rate (/min)	85, (73–99)	82, (72–95)	89, (75–104)
Respiratory rate (/min)	16, (14–18)	16, (14–17)	16, (14–18)
First prehospital glucose, (mmol/l)	2.8, (1.6–3.6)	1.8, (1.2–2.8)	3.6, (3.2–3.8)
GCS	12, (0–15)	9, (0–15)	14, (0–15)
Hypertension (%)	18, (17–18)	23, (22–24)	10.2, (9.4–11.0)
Atrial fibrillation (%)	2.1, (1.9–2.4)	2.3, (2.0–2.7)	1.9, (1.5–2.2)
Chronic alcoholism (%)	1.8, (1.5–2.0)	0.7, (0.6–1.0)	3.2, (2.7–3.7)
Chronic renal failure (%)	1.8, (1.6–2.1)	2.8, (2.5–3.2)	0.5, (0.3–0.7)
Chronic liver failure (%)	0.3, (0.2–0.4)	0.3, (0.2–0.4)	0.3, (0.2–0.5)
Dementia (%)	1.5, (1.3–1.7)	1.7, (1.4–2.0)	1.3, (1.0–1.7)
Glucose (iv/po) administered (%)	49.3, (48.4–50.1)	71.9, (70.9–72.9)	17.5, (16.5–18.6)
Transported to hospital (%)	48.8, (48.0–49.7)	32.6, (31.5–33.6)	71.6, (70.4–72.8)
A	2.6, (2.2–3.0)	2.1, (1.6–2.8)	2.8, (2.4–3.4)
B	7.3, (6.7–7.9)	5.9, (5.0–6.9)	8.2, (7.4–9.1)
C	60.9, (59.7–62.1)	65.3, (63.4–57.2)	58.1, (56.6–59.7)
D	29.3, (28.2–30.4)	26.7, (25.0–28.5)	30.9, (29.5–32.4)
Overall mortality rate of plasma glucose ≤3.9 mmol/l (%)	11.3, (10.8–11.9)	9.8, (9.2–10.5)	13.4, (12.6–14.4)
Overall mortality rate of plasma glucose ≤3.0 mmol/l (%)	10.4, (9.7–11.1)	8.6, (8.0–9.4)	20.1, (17.9–22.6)

Abbreviations: GCS = Glasgow Coma Score, A, B, C, and D = dispatch codes. A = highest priority to D = lowest priority

Data are presented as median and interquartile range except for sex and transportation, which are presented as percentage with 95% confidence interval

### Aetiology

A possible aetiological cause that may have caused a hypoglycaemic event in patients that were transferred to hospital was found in 3685 [95.6%, (CI95% 94.9–96.2)] and in the serious hypoglycaemia group 880 [96.7%, (CI95% 95.3–97.7)]. Only one possible aetiological cause that was identified as the hospital admission cause possibly linked to a hypoglycaemic event were recorded in 55.1% (CI95% 53.6–56.7) cases without diabetes. When concomitant possible aetiological causes were studied two concomitant possible causes were in 30.7% (CI95% 29.3–32.2) of cases and three or more possible causes in 9.4% (CI95% 8.5–10.3) of cases. When serious hypoglycaemia was present 43.9% (CI95% 40.7–47.1) had only one possible aetiological cause, 31.9% (CI95% 28.9–35.0) identified two, and 20.3% (CI95% 17.8–23.1) identified three or more hospital treatment causes that potentially caused a hypoglycaemic event.

The aetiological causes for hospital stay with potential association of hypoglycaemia are presented in Table 2. Aetiological causes with only one possible aetiological cause found having a potential association to hypoglycaemia are presented in Additional file 2. Malnutrition included conditions such as eating disorders, short-bowel syndrome, dysphagia, nausea, temporary physical or mental invalidity, or due to too hectic life schedule. Infections were most commonly gastroenteritis, sepsis or pneumonia. In some infection-related cases peritonitis and human immunosuppressive virus (HIV) were identified as possible cause of serious hypoglycaemia. Acute sympathetic nervous system activation was common in mild hypoglycaemic cases without diabetes, but not when serious hypoglycaemia was present.

**Table 2:** Non-diabetic aetiological causes by all (plasma glucose ≤3.9 mmol/l) and serious hypoglycaemia (plasma glucose ≤3.0 mmol/l)

	≤3.9 mmol/l	≤3.0 mmol/l
N	3856	910
Alcohol abuse	41.4, (39.8–42.9)	42.2, (39.0–45.4)
Hypothermia	17.2, (16.0–18.4)	27.4, (24.6–30.4)
Malnutrition	17.0, (15.8–18.2)	25.1, (22.4–28.0)
Intoxication	13.5, (12.4–14.6)	11.7, (9.7–13.9)
Infections	14.3, (13.3–15.5)	20.1, (17.6–22.8)
Acute sympathetic nervous system activation and peripheral vasoconstriction	5.6, (4.9–6.3)	3.9, (2.8–5.3)
Renal failure	4.3, (3.7–5.0)	9.0, (7.3–11.1)
Liver failure	3.1, (2.6–3.7)	8.5, (6.8–10.5)
Congestive heart failure	2.1, (1.7–2.6)	2.9, (1.9–4.2)
Out-of-hospital cardiac arrest	0.5, (0.3–0.8)	1.1, (0.6–2.0)
Neurological disorders	14.8, (13.7–16.0)	9.2, (7.5–11.3)
Endocrinological disorders	0.7, (0.5–1.0)	2.1, (1.3–3.3)
Malignancies	3.0, (2.5–3.6)	5.8, (4.5–7.6)
Unspecified fatigue, unspecified dizziness	1.3, (1.0–1.7)	0.4, (0.1–1.2)
Unknown	4.0, (3.4–4.7)	2.4, (1.6–3.7)

Data are presented as percentage with 95% confidence interval

In the patients with intoxication as hospital treatment cause, the substance causing intoxication was identified from hospital records in 92.8% (CI95% 90.3–94.7) of the cases. Most common substances were recreational drugs [48.6%, (CI95% 44.3–52.8)], benzodiatsepins [34.0% (CI95% 30.1–38.1)], and analgesics [17.3%, (CI95% 14.3–20.7)]. Antihyperglycaemic drugs were identified as the cause of intoxication in only 7.8%, (CI95% 5.8–10.4) of cases. Neurological disorders were a possible cause of the hypoglycaemia episode in approximately one tenth of cases. These consisted mostly of epilepsy [31.2%, (CI95% 27.5–35.0)], concussion [26.7%, (CI95% 23.3–30.5)], and stroke [13.2%, (CI95% 10.7–16.2)]. Malignancies, liver failure, renal failure, congestive heart failure and out-of-hospital cardiac arrest (OHCA) were possible contributing causes of hypoglycaemia in only 10.0% (CI95% 9.1–11.0) cases (Table 2).

There were 0.7%, (CI95% 0.5–1.0) endocrine disorders that caused hypoglycaemia in our study population. These consisted of three cases of insulinoma, one case of hyperinsulinism, thirteen cases of hypopituitarism and nine cases of Addison’s disease. The other hospital treatment causes possibly related to the development of a hypoglycaemic episode were listed as a symptom of hypoglycaemia and hence an unspecified event. These conditions presented as unspecified fatigue or unspecified dizziness.

### Outcome

**Fig. 2:**
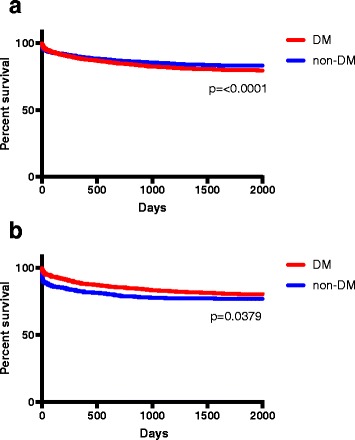
Kapplan-Mayer survival curves comparing diabetic and non-diabetic mortality. Hypoglycaemic cases (≤3.9 mmol/l) is (**a)** and serious hypoglycaemic cases (≤3.0 mmol/l) is (**b)**

Patients without diabetes had significantly lower survival rates, when serious hypoglycaemia (≤3.0 mmol/l) was present, *p* = 0.0397. However, patients with diabetes had lower survival rate, when all hypoglycaemic cases (≤3.9 mmol/l) where considered, p = < 0.0001. Mortality rate varied greatly between different aetiological possible causes of hypoglycaemia showing highest mortality with renal failure, liver failure, congestive heart failure, malignancies, and sudden out-of-hospital cardiac arrest (OHCA) (Table 3 and Fig. 2).

**Table 3:** Mortality by aetiology of hypoglycaemic patients without diabetes (plasma glucose ≤3.9 mmol/l)

Aetiology	N	24 h %, (95% CI)	30 days %, (95% CI)	1 year %, (95% CI)
Alcohol abuse	1594	1.0, (0.6–1.6)	2.2, (1.6–3.0)	6.0, (4.9–7.2)
Hypothermia	662	5.6, (4.1–7.6)	10.4, (8.3–13.0)	14.8, (12.3–17.7)
Malnutrition	650	1.8, (1.1–3.2)	7.8, (6.0–10.1)	13.2, (10.8–16.0)
Neurological disorders	571	1.4, (0.7–2.7)	4.7, (3.3–6.8)	8.8, (6.7–11.4)
Acute sympathetic nervous system activation and peripheral vasoconstriction	214	3.7, (1.9–7.2)	5.6, (3.2–9.6)	9.8, (6.5–14.5)
Infection	544	5.2, (3.7–7.4)	12.8, (10.3–15.9)	20.1, (16.9–23.6)
Intoxication	517	0.6, (0.2–1.7)	1.7, (0.9–3.3)	3.7, (2.4–5.7)
Renal failure	166	16.9, (11.9–23.3)	36.8, (29.8–44.3)	48.8, (41.3–56.3)
Liver failure	119	19.3, (13.2–27.3)	42.9, (34.3–51.8)	50.4, (41.6–59.3)
Malignancies	116	12.9, (8.0–20.2)	38.8, (30.4–47.9)	51.7, (42.7–60.6)
Congestive heart failure	81	13.6, (7.8–22.7)	29.6, (20.8–40.3)	46.9, (36.4–57.7)
Unspecified fatigue, dizziness	50	0.0, (0.0–7.1)	0.0, (0.0–7.1)	4.0, (1.1–13.5)
Endocrine disorders	27	0.0, (0.0–12.5)	0.0, (0.0–12.5)	7.4, (2.1–23.4)
Out-of-hospital cardiac arrest and subsequent resuscitation	20	50.0, (29.9–70.1)	55.0, (34.2–74.2)	60.0, (38.7–78.1)
Unknown	154	0.0, (0.0–2.4)	0.7, (0.1–3.6)	2.0, (0.7–5.6)
Not transferred to hospital	1552	1.0, (0.6–1.6)	1.8, (1.3–2.6)	4.1, (3.2–5.2)

*N* = 3856. Data are presented as percentage with 95% confidence interval

### Risk factor associated with mortality

The result of univariable and multivariable logistic regression analysis for risk factors associated with mortality are presented in Table 4. After adjusting for confounding factors multivariate analysis showed that age, higher priority dispatch codes, and plasma glucose rate had a strong association to mortality. Logistic regression analysis for mortality of alcohol abuse, hypothermia, malnutrition, infections, intoxications, and neurological disorders, can be seen in Additional files 3 and 4.

**Table 4:** Univariate and multivariate logistic regression analysis for mortality, N = 3856

		Odds ratio, (CI95%)	*p*-value
Univariate	Sex (male)	0.86, (0.72–1.03)	0.110
	Age	1.05, (1.04–1.06)	< 0.001
	Plasma glucose ≤3.9 mmol/l	0.65, (0.57–0.74)	< 0.001
	Plasma glucose ≤3.0 mmol/l	1.80, (1.49–2.18)	< 0.001
	A	2.53, (1.61–3.91)	< 0.001
	B	1.58, (1.15–2.17)	0.005
	C	1.00, (0.82–1.22)	0.968
	D	reference	
Multivariate	Sex (male)	1.04, (0.85–1.26)	0.723
	Age	1.05, (1.05–1.06)	< 0.001
	Plasma glucose ≤3.9 mmol/l	reference	
	Plasma glucose ≤3.0 mmol/l	1.72, (1.41–2.09)	< 0.001
	A	3.85, (2.34–6.23)	< 0.001
	B	2.44, (1.72–3.45)	< 0.001
	C	1.47, (1.18–1.83)	< 0.001
	D	reference	

A, B, C, and D = dispatch codes. A = highest priority to D = lowest priority

## Discussion

Our study found that hypoglycaemic cases without diabetes was common among hypoglycaemic EMS cases. Alcohol abuse, hypothermia, and malnutrition were the most common possible etiological causes associated with a hypoglycaemic episode. Mortality correlated with age, higher priority dispatch codes, and plasma glucose rate with multivariate logistic regression analysis. Some etiological subgroups carry a markedly high mortality rate.

Possible hypoglycaemia related etiological causes encountered in the EMS differed from previously found causes in hospitalized patients. Alcohol abuse, intoxication, hypothermia, and malnutrition were overly presented in the EMS population compared to possible hypoglycaemia aetiologies found in the hospital ward: renal failure, liver failure, sepsis, congestive heart failure, and malignancies [3]. A possible explanation may be that these EMS found aetiologies are the ones most frequently encountered by EMS. As these possible causes of hypoglycaemia are relatively benign, the patients are often left home or have only short hospital admissions. In consequence, these aetiologies are less prevalent in studies of hospital study populations [9–11].

The incidence for hypoglycaemia in patients without diabetes was relatively high in our study compared to other studies [2, 3, 12–16]. Our study cohort was large, population based and used EMS data records compared to most of previous studies [3, 12, 13, 15, 16]. In the largest previous study by Sako et al. [14] the hypoglycaemia was defined by extracting diagnosis codes from patient records whereas we used EMS patient records and plasma glucose values. This can explain higher incidence in the current study. Earlier EMS record-based studies by Parsaik et al. [2] and Tjusimoto et al. [15], did not address the severity of hypoglycaemia defined by blood glucose level. Thus, comparisons with these studies is difficult. The overall mortality rate in the current study was in accordance to previous findings [3, 12, 15, 16]. Only Sako et al. [14] from previous studies did logistic regression analysis to define risk factor that correlated to mortality. We also used logistic regression analysis to determine the risk factors for mortality.

The most common possible alcohol-induced hypoglycaemia mechanisms are alcohol-induced fasting hypoglycaemia, inhibition of hepatic gluconeogenesis, down regulation of counter-regulatory hormones, and decreased glucose release form liver. Hypothermia causes underestimation of blood glucose value due to capillary constriction and disturbs glucose homeostasis directly [11, 16].

Microcirculatory alteration may be present during hypoglycaemic incidents due to sympathetic nervous system activation, although not affecting significantly hemodynamics, causing mild pseudohypoglycaemia [17], This phenomenon has been observed in critically ill patients [18]. In addition, blood glucose levels decrease locally when impaired tissue perfusion in the lower capillary flow leads to extended time for glucose to pass the extremity area and time for greater glucose extraction [8]. Hence actual local hypoglycaemia development may in some degree be also present. However, people experiencing acute gastrointestinal, chest, or back pain may just simply be feeling so unwell and hence food intake is diminished temporarily.

Hypoglycaemia may be related to neurological disorders such as epilepsy or concussion [19, 20]. Infection-related possible hypoglycaemia episodes are often related to sepsis and pneumonia, but also milder infections such as urinary, respiratory or skin/soft tissue infections [21]. Many non-antihyperglycaemic drugs and recreational drugs can induce hypoglycaemia thought with quite low evidence [16, 22–24].

When liver failure, congestive heart failure, OCHA, renal failure, or malignancies constitute the likely contributing cause for hypoglycaemia, then mortality is high in patients without diabetes. Renal failure affects hypoglycaemia development through decreased insulin clearance, diminished gluconeogenesis, and poor caloric intake. [3, 4, 12, 25]. In cases of congestive heart failure and cardiogenic shock, hypoglycaemia may be related to secondary liver failure [26], altered microcirculation, and increased tissue perfusion time due to oedema [17]. In malignancy-induced hypoglycaemia the tumour might secrete cytokines, utilize glucose itself, or cause loss of appetite [3]. These conditions may per se cause hypoglycaemia, but hypoglycaemia in these cases may often signal an underlying severe illness.

Clinicians should keep in mind that hypoglycaemia incidents seen in patients without diabetes carry a poor outcome, especially when serious hypoglycaemia is encountered. Hypoglycaemia may be a sign of underlying critical illness. Further prospective and case-control studies should be made to better define possible aetiologies, incidence and prevalence for hypoglycaemia as an underlying sign for critical illness.

The main limitation of our study was the retrospective study design. Also, the study data was gathered from a patient record system and thus, the validation of the data is difficult. However, as the data used is actually a primary source data, validation may not be needed. Other limitations were that plasma glucose was not measured for study purpose, but according to local protocol. This may have caused bias into the results. In EMS data or hospital records, there was limited information on the medications used by patients. Especially, in the hypoglycaemia cases without diabetes included in our study, we did not know patients’ current medication at the time when the hypoglycaemic episode occurred. For instance, it is unknown whether patients were taking fluoroquinolones or other drugs potentially having a role in the hypoglycaemic episode. The time of patients’ meals could also not be determined. Unknown periods of fasting might have affected the results. Blood glucose values by EMS personnel were mainly taken from capillary samples, although in a minority of cases they were taken from venous and arterial lines. This may have affected the accuracy of results [18, 27]. EMS data records of current medical conditions was recorded from information of discretion of the patient or the next of kin as well from information gathered from social security card. Human errors may have caused bias to these results. There is evidence suggesting that chronic cigarette smoking may contribute to the development of hypoglycaemia [28]. In our study, we could not determine the patients’ smoking status. Hence, we could not define the impact of smoking on hypoglycaemia. Finally, restrictions on data extraction from the electronical hospital patient record system required manual data collection on possible aetiological causes by only one author. This may have caused a bias.

## Conclusion

We conclude that hypoglycaemic EMS cases without diabetes is frequently observed among hypoglycaemic EMS cases. The most common causes for it are alcohol abuse, hypothermia, and malnutrition. Mortality correlated with age, higher priority dispatch codes, and plasma glucose rate. Some of the etiological subgroups carried a markedly high mortality rate.

## Additional files

Additional file 1: Aetiological causes, when only one possible aetiological cause was present. (DOCX 62 kb)

Additional file 2: Mortality by aetiology of serious hypoglycaemic patients without diabetes (plasma glucose ≤3.0 mmol/l). *N* = 910. (DOCX 85 kb)

Additional file 3: Univariate and multivariate logistic regression analysis of mortality for alcohol abuse (*n* = 1594), hypothermia (*n* = 662), and malnutrition (*n* = 650). (DOCX 91 kb)

Additional file 4: Univariate and multivariate logistic regression analysis of mortality of infections (*n* = 544), intoxications (*n* = 517), and neurologic disorders (*n* = 571). (DOCX 95 kb)

## Abbreviations

A: highest priority dispatch code
ADA: American diabetes association
B: second highest priority dispatch code
C: second lowest dispatch code
D: lowest dispatch code
EMA: European medicines agency
EMS: emergency medical services
GCS: Glasgow coma scale
HIV: human immunosuppressive virus
OHCA: out of hospital cardiac arrest
