# Supplementary material for: Hypoglycaemia without diabetes encountered by emergency medical services: a retrospective cohort study
Source: Scand J Trauma Resusc Emerg Med. 2018 Feb 1;26:12. doi: 10.1186/s13049-018-0480-7 (PMC5796568; doi:10.1186/s13049-018-0480-7)
Supplement: Supplementary file 1 — Aetiological causes, when only one possible aetiological cause was present. (DOCX 62 kb) [file 13049_2018_480_MOESM1_ESM.docx]

|  | Hypoglycaemia  (≤3.9 mmol/l) | Serious hypoglycaemia (≤3.0 mmol/l) |
| --- | --- | --- |
| N | 2124 | 424 |
| Alcohol abuse | 34.1, (32.1-36.2) | 31.6, (27.4-36.2) |
| Hypothermia | 1.5, (1.0-2.1) | 1.7, (0.7-3.4) |
| Malnutrition | 11.4, (10.1-12.8) | 16.0, (12.8-19.9) |
| Intoxication | 8.8, (7.7-10.1) | 9.7, (7.2-12.9) |
| Infections | 9.0, (7.8-10.2) | 12.0, (9.3-15.5) |
| Acute sympathetic nervous system activation and peripheral vasoconstriction | 10.1, (8.9-11.4) | 8.0, (5.8-11.0) |
| Renal failure | 0.6, (0.4-1.0) | 0.2, (0.0-1.5) |
| Liver failure | 0.4, (0.2-0.8) | 0.7, (0.1-2.2) |
| Congestive heart failure | 0.7, (0.4-1.2) | 0.5, (0.0-1.8) |
| Out-of-hospital cardiac arrest | 0.9, (0.6-1.5) | 2.4, (1.2-4.4) |
| Neurological disorders | 16.6, (15.1-18.3) | 10.9, (8.2-14.2) |
| Endocrinological disorders | 0.4, (0.2-0.8) | 2.1, (1.1-4.1) |
| Malignancies | 1.2, (0.8-1.7) | 2.4, (1.2-4.4) |
| Unspecified fatigue, unspecified dizziness | 1.8, (1.3-2.5) | 0.7, (0.1-2.2) |
| Unknown | 7.3, (6.2-8.4) | 5.2, (3.4-7.8) |
