# Supplementary material for: Hypoglycaemia without diabetes encountered by emergency medical services: a retrospective cohort study
Source: Scand J Trauma Resusc Emerg Med. 2018 Feb 1;26:12. doi: 10.1186/s13049-018-0480-7 (PMC5796568; doi:10.1186/s13049-018-0480-7)
Supplement: Supplementary file 2 — Mortality by aetiology of serious hypoglycaemic patients without diabetes (plasma glucose ≤3.0 mmol/l). N = 910. (DOCX 85 kb) [file 13049_2018_480_MOESM2_ESM.docx]

| Aetiology | N | 24 hours %, (95%CI) | 30 days %, (95%CI) | 1 year %, (95 %CI) |
| --- | --- | --- | --- | --- |
| Alcohol abuse | 384 | 2.6, (1.4-4.8) | 5.5, (3.6-8.3) | 9.6, (7.0-13.0) |
| Hypothermia | 253 | 9.1, (6.1-13.3) | 15.8, (11.8-20.8) | 21.7, (17.1-27.2) |
| Malnutrition | 228 | 2.6, (1.1-5.8) | 11.8, (8.2-16.7) | 18.9, (14.3-24.5) |
| Neurological disorders | 84 | 3.6, (0.8-10.4) | 10.7, (5.5-19.3) | 16.7, (10.1-26.2) |
| Acute sympathetic nervous system activation and peripheral vasoconstriction | 35 | 8.6, (2.2-23.1) | 14.3, (5.8-29.9) | 22.9, (11.8-39.3) |
| Infection | 183 | 9.8, (6.2-15.1) | 20.2, (15.0-26.7) | 29.0, (22.9-35.9) |
| Intoxication | 106 | 1.9, (0.1-7.0) | 6.6, (3.0-13.2) | 10.4, (5.7-17.8) |
| Renal failure | 82 | 23.2, (15.3-33.5) | 46.3, (36.0-57.1) | 57.3, (46.5-67.5) |
| Liver failure | 77 | 26.0, (17.4-36.8) | 46.8, (36.0-57.8) | 50.7, (39.7-61.5) |
| Malignancies | 53 | 13.2, (6.2-25.2) | 35.9, (24.3-49.3) | 39.6, (27.6-53.1) |
| Congestive heart failure | 26 | 26.9, (13.5-46.3) | 46.2, (28.8-64.6) | 65.4, (46.1-80.7) |
| Unspecified fatigue, Unspecified dizziness | 4 | 0.0, (0.0-0.5) | 0.0, (0.0-0.5) | 0.0, (0.0-0.5) |
| Endocrine disorders | 19 | 0.0, (0.0-19.8) | 0.0, (0.0-19.8) | 5.3, (0.0-26.5) |
| Out-of-hospital cardiac arrest and subsequent resuscitation | 10 | 60.0, (31.2-83.3) | 60.0, (31.2-83.3) | 60.0, (31.2-83.3) |
| Unknown | 22 | 0.0, (0.0-17.6) | 4.6, (0.0-23.5) | 4.6, (0.0-23.5) |
| Not transferred to hospital | 173 | 3.5, (1.4-7.5) | 6.4, (3.5-11.1) | 8.1, (4.8-13.2) |
