# Supplementary material for: Hypoglycaemia without diabetes encountered by emergency medical services: a retrospective cohort study
Source: Scand J Trauma Resusc Emerg Med. 2018 Feb 1;26:12. doi: 10.1186/s13049-018-0480-7 (PMC5796568; doi:10.1186/s13049-018-0480-7)
Supplement: Supplementary file 3 — Univariate and multivariate logistic regression analysis of mortality for alcohol abuse (n = 1594), hypothermia (n = 662), and malnutrition (n = 650). (DOCX 91 kb) [file 13049_2018_480_MOESM3_ESM.docx]

|  | Alcohol abuse |  |  | Hypothermia |  |  | Malnutrition |  |  |
| --- | --- | --- | --- | --- | --- | --- | --- | --- | --- |
| Univariate | Sex (male) | 1.22, (0.85-1.77) | 0.295 | Sex (male) | 0.75, (0.50-1.14) | 0.171 | Sex (male) | 0.92, (0.61-1.37) | 0.683 |
|  | Age | 1.03, (1.02-1.04) | <0.001 | Age | 1.04, (1.03-1.05) | <0.001 | Age | 1.05, (1.04-1.06) | <0.001 |
|  | Plasma glucose (≤3.9 mmol/l) | 0.78, (0.63-0.99) | 0.034 | Plasma glucose (≤3.9 mmol/l) | 0.58, (0.45-0.75) | <0.001 | Plasma glucose (≤3.9 mmol/l) | 0.65, (0.48-0.87) | 0.003 |
|  | Plasma glucose (≤3.0 mmol/l) | 1.44, (1.03-2.00) | 0.031 | Plasma glucose (≤3.0 mmol/l) | 2.04, (1.38-3.00) | <0.001 | Plasma glucose (≤3.0 mmol/l) | 1.81, (1.21-2.71) | 0.004 |
|  | A | 3.49, (1.48-7.63) | 0.003 | A | 5.56, (2.41-12.90) | <0.001 | A | 5.47, (0.21-140.00) | 0.233 |
|  | B | 1.94, (1.03-3.49) | 0.032 | B | 3.53, (1.78-7.09) | <0.001 | B | 3.65, (1.60-8.14) | 0.002 |
|  | C | 1.40, (1.00-1.99) | 0.057 | C | 1.80, (1.08-3.13) | 0.029 | C | 1.22, (0.80-1.90) | 0.361 |
|  | D | reference |  | D | reference |  | D | reference |  |
| Multivariate | Sex (male) | 1.18, (0.82-1.74) | 0.376 | Sex (male) | 1.07, (0.69-1.68) | 0.764 | Sex (male) | 1.05, (0.68-1.62) | 0.827 |
|  | Age | 1.03, (1.02-1.04) | <0.001 | Age | 1.04, (1.03-1.05) | <0.001 | Age | 1.05, (1.03-1.06) | <0.001 |
|  | Plasma glucose (≤3.9 mmol/l) | reference |  | Plasma glucose (≤3.9 mmol/l) | reference |  | Plasma glucose (≤3.9 mmol/l) | reference |  |
|  | Plasma glucose (≤3.0 mmol/l) | 1.34, (0.95-1.86) | 0.096 | Plasma glucose (≤3.0 mmol/l) | 1.73, (1.16-2.58) | 0.007 | Plasma glucose (≤3.0 mmol/l) | 1.76, (1.16-2.66) | 0.008 |
|  | A | 3.16, (1.32-6.97) | 0.006 | A | 4.61, (1.96-10.80) | <0.001 | A | 6.35, (0.25-163.00) | 0.195 |
|  | B | 1.82, (0.96-3.30) | 0.054 | B | 3.02, (1.50-6.13) | 0.002 | B | 3.23, (1.40-7.27) | 0.005 |
|  | C | 1.39, 80.99-1.97) | 0.062 | C | 1.70, (1.01-2.96) | 0.053 | C | 1.09, (0.70-1.72) | 0.693 |
|  | D | reference |  | D | reference |  | D | reference |  |

Abbreviations: A, B, C, and D=dispatch codes. A=highest priority to D= lowest priority.
