# Supplementary material for: Hypoglycaemia without diabetes encountered by emergency medical services: a retrospective cohort study
Source: Scand J Trauma Resusc Emerg Med. 2018 Feb 1;26:12. doi: 10.1186/s13049-018-0480-7 (PMC5796568; doi:10.1186/s13049-018-0480-7)
Supplement: Supplementary file 4 — Univariate and multivariate logistic regression analysis of mortality of infections (n = 544), intoxications (n = 517), and neurologic disorders (n = 571). (DOCX 95 kb) [file 13049_2018_480_MOESM4_ESM.docx]

|  | Infections | OR, (CI95%) | p-value | Intoxications | OR, (CI95%) | p-value | Neurological disorders | OR, (CI95%) | p-value |
| --- | --- | --- | --- | --- | --- | --- | --- | --- | --- |
| Univariate | Sex (male) | 0.69, (0.47-1.02) | 0.061 | Sex (male) | 1.69, (0.84-3.62) | 0.158 | Sex (male) | 0.85, (0.53-1.36) | 0.489 |
|  | Age | 1.04, (1.03-1.05) | <0.001 | Age | 1.04, (1.02-1.06) | <0.001 | Age | 1.05, (1.04-1.07) | <0.001 |
|  | Plasma glucose (≤3.9 mmol/l) | 0.75, (0.58-0.98) | 0.036 | Plasma glucose (≤3.9 mmol/l) | 0.56, (0.37-0.87) | 0.007 | Plasma glucose (≤3.9 mmol/l) | 0.61, (0.41-0.94) | 0.020 |
|  | Plasma glucose (≤3.0 mmol/l) | 1.47, (0.98-2.19) | 0.061 | Plasma glucose (≤3.0 mmol/l) | 2.39, (1.16-4.74) | 0.014 | Plasma glucose (≤3.0 mmol/l) | 1.77, (0.97-3.12) | 0.055 |
|  | A | 1.69, (0.56-4.66) | 0.323 | A | 3.40, (0.84-14.70) | 0.085 | A | 2.76, (0.77-9.03) | 0.100 |
|  | B | 1.65, (0.85-3.15) | 0.134 | B | 1.57, (0.43-6.38) | 0.500 | B | 1.33, (0.56-3.22) | 0.514 |
|  | C | 1.10, (0.71-1.73) | 0.665 | C | 1.22, (0.45-4.25) | 0.724 | C | 1.19, (0.62-2.49) | 0.624 |
|  | D | reference |  | D | reference |  | D | reference |  |
| Multivariate | Sex (male) | 0.84, (0.55-1.29) | 0.433 | Sex (male) | 1.80, (0.86-3.98) | 0.129 | Sex (male) | 1.08, (0.64-1.83) | 0.770 |
|  | Age | 1.04, (1.03-1.05) | <0.001 | Age | 1.04, (1.02-1.06) | <0.001 | Age | 1.05, 81.04-1.07) | <0.001 |
|  | Plasma glucose (≤3.9 mmol/l) | reference |  | Plasma glucose (≤3.9 mmol/l) | reference |  | Plasma glucose (≤3.9 mmol/l) | reference |  |
|  | Plasma glucose (≤3.0 mmol/l) | 1.39, (0.91-2.10) | 0.126 | Plasma glucose (≤3.0 mmol/l) | 2.18, (1.04-4.38) | 0.032 | Plasma glucose (≤3.0 mmol/l) | 1.77, (0.96-3.15) | 0.056 |
|  | A | 1.55, (0.51-4.31) | 0.415 | A | 2.71, (0.65-12.00) | 0.169 | A | 2.67, (0.74-8.79) | 0.113 |
|  | B | 1.44, (0.72-2.82) | 0.291 | B | 1.43, (0.39-5.87) | 0.592 | B | 1.41, (0.59-3.43) | 0.437 |
|  | C | 1.04, (0.66-1.64) | 0.878 | C | 1.18, (0.43-4.12) | 0.773 | C | 1.19, (0.61-2.50) | 0.625 |
|  | D | reference |  | D | reference |  | D | reference |  |

Abbreviations: A, B, C, and D=dispatch codes. A=highest priority to D= lowest priority.
